# The thermo-optic relevance of Ho^3+^ in fluoride microcrystals embedded in electrospun fibers

**DOI:** 10.1039/d0ra08696g

**Published:** 2020-11-11

**Authors:** Yan Zhang, Zelin Gao, Yue Li, Edwin Yue Bun Pun, Hai Lin

**Affiliations:** School of Textile and Material Engineering, Dalian Polytechnic University Dalian 116034 China lhai8686@yahoo.com; Department of Electrical Engineering and State Key Laboratory of Terahertz and Millimeter Waves, City University of Hong Kong Tat Chee Avenue Kowloon Hong Kong China

## Abstract

Na(Y_1−*x*−*y*_Ho_*x*_Yb_*y*_)F_4_/PAN (NYF-HY/PAN) composite fibers were synthesized using an electrospinning method, and the sub-micron crystals embedded in the fibers had complete hexagonal crystal structures. Under 977 nm laser excitation, strong green and red up-conversion (UC) emission that originated from flexible fibers were due to the radiative transitions (^5^F_4_, ^5^S_2_) → ^5^I_8_ and ^5^F_5_ → ^5^I_8_ of Ho^3+^, respectively. The effective green fluorescence emission (539 and 548 nm) can be applied to micro-domain non-contact temperature measurements, realizing rapid and dynamic temperature acquisition in a complex environment without destroying the temperature field. In the temperature range of 313–393 K, the absolute and relative sensitivity of the fibers are 0.00373 K^−1^ and 0.723% K^−1^, respectively, which indicates that the NYF-HY/PAN composite fibers have good thermal sensitivity. Composite fibers in which crystallites are embedded have superior properties, with great stability, high sensitivity, and excellent flexibility, providing a reliable reference for developing temperature-sensing materials for the biomedical field.

## Abstract 2

The morphology of electrospun fibers embedded with microcrystals and the relationship between sensitivity and temperature based on green up-conversion emission are studied.

## Graphical abstract

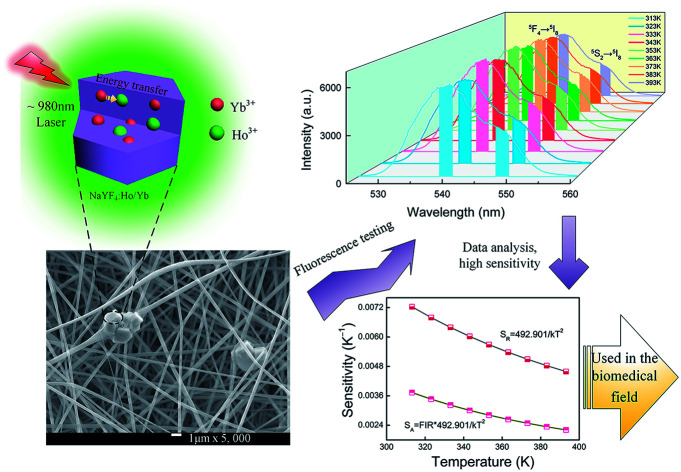

## 1. Introduction

It is generally known that temperature sensors play a crucial role in most applications, such as biology, agriculture, military and medicine.^1,2^ Recently, non-contact temperature measurement has received widespread attention because it is able to meet the requirements for use in dangerous applications such as strong electromagnetic, high voltage and non-contact environments.^3–7^ A suitable choice for non-contact temperature measurement is the temperature measurement feedback method based on the fluorescence intensity ratio (FIR), which avoids spectral loss and excitation source fluctuation, and its sensitivity and accuracy are higher than traditional ways of measuring temperature.^8–15^ Rare Earth (RE) ion doped materials are widely applied in non-contact temperature control measurement because their thermal coupling energy levels respond to changes in laser power and temperature.^16–23^ By measuring the fluorescence intensity ratio of the thermal coupling energy levels of the RE doped materials, the temperature changes around the fluorescent probe can be monitored.^24–31^ Therefore, RE ion doped temperature sensing materials are considered to be a very promising for temperature detection materials.^32–40^

Among the trivalent RE ions, Ho^3+^ has plentiful energy levels and obvious luminescence characteristics in the visible light region,^41,42^ and it has the ability to obtain intense optical light by the sensitization of other RE ions.^43–45^ Meanwhile, the use of Ho^3+^ as a potential candidate for temperature sensing has been confirmed in RE, and thermal-coupled levels of Ho^3+^ ions, ^5^F_4_ and ^5^S_2_, ^5^S_2_ and ^5^F_5_, ^5^F_3_ and ^3^K_8_, and ^5^F_2,3_/^3^K_8_ and ^5^G_6_/^5^F_1_ have all been investigated in temperature measurement applications.^44–48^ When Yb^3+^ is introduced, the effective luminescence originated by the previously mentioned thermal-coupled energy levels of Ho^3+^ can be applied for temperature sensing.^49,50^ Among the numerous forms of matrix materials, powder, single crystals, and glass have the disadvantages of requiring molding materials and having a constant shape when used as temperature sensing materials. Therefore, flexible materials for microdomain complexity thermal reaction could be adopted. Flexibility is a characteristic of polymer materials,^51^ however its rich hydroxyl structure is fatal to fluorescence emission from thermally coupled energy levels. Fortunately, composite fibers of organic–inorganic combination can perfectly solve this defect, and realize effective fluorescence emission for thermal reaction measurement.

In the present study, a series of Ho^3+^/Yb^3+^ co-doped NaYF_4_/PAN composite fibers were prepared by an electrospinning technique. It is confirmed that the microcrystals (MCs) of the hexagonal stable crystal phase are intact in the fibers and have complete crystalline functions. The UC performance of the samples is explored in detail, and the influence of Yb^3+^ concentration co-doping on its UC performance is investigated and the energy transfer processes between Ho^3+^ and Yb^3+^ ions are determined. Furthermore, the green UC emission intensity ratio from thermal correlation levels ^5^F_4_ and ^5^S_2_ is studied by a FIR technique, which is a function of temperature in the range of 313–393 K. It is found that the flexible fibers have great thermal sensitivity, which indicates that the NYF-HY/PAN fiber has prospects for a broad application in the field of temperature sensors.

## 2. Experimental

### 2.1 Materials and methods

#### 2.1.1 Synthesis of NYF-HY MCs

The Ho^3+^/Yb^3+^ co-doped NaYF_4_ (NYF) MCs were prepared by hydrothermal synthesis. Before preparing the MCs, high-purity powders of Y_2_O_3_ (99.99%), Yb_2_O_3_ (99.99%) and Ho_2_O_3_ (99.99%) were dissolved in hydrochloric acid (HCl, AR) to obtain the corresponding RECl_3_·6H_2_O (RE = Y, Yb or Ho). Subsequently, 3 mmol of RECl_3_·6H_2_O (RE = Y, Yb or Ho with designated molar ratios) was dissolved in 10 mL of deionized water and stirred uniformly. Then a mixed aqueous solution of 0.63 g of citric acid (H_3_Cit·H_2_O, AR) and 0.36 g of sodium hydroxide (NaOH, AR) were added to obtain 30 mL of mixed solution S1. In the S1 solution, complexes of RE^3+^ ions and Cit^3−^ radicals take the form of a white suspended precipitate as the reaction proceeds. Next, to obtain a concentration ratio of the F^−^/RE^3+^ of 7, the NaF (AR) aqueous solution was added to S1 while stirring, and its pH was adjusted to 6–7 to obtain 60 mL of mixed solution S2. The solution S2 was transferred into a 100 mL stainless steel Teflon-lined autoclave, which was operated at 200 °C for 12 h under autogenous pressure in the constant temperature heating/drying oven, and then cooled to room temperature. Finally, it was washed three times, alternating deionized water and ethanol, to obtain the required MCs, and then dried at 70 °C for 12 h in the drying oven. The MCs can be described as Na(Y_1−*x*−*y*_Ho_*x*_Yb_*y*_)F_4_, where *x* = 0.01*m*, *y* = 0.01*n*, (*m*,*n*) = (0,0) for a matrix sample, (*m*,*n*) = (1,0) for Ho^3+^ ions single-doped sample, and (*m*,*n*) = (1,1), (1,2), (1,3), (1,4) for Ho^3+^ and Yb^3+^ ions co-doped samples. For a better demonstration, the corresponding MC samples are labeled as NYF-H_0_Y_0_, NYF-H_1_Y_0_, NYF-H_1_Y_1_, NYF-H_1_Y_2_, NYF-H_1_Y_3_, NYF-H_1_Y_4_.

#### 2.1.2 Preparation of NYF-HY/PAN composite fibers

**Fig. 1:**
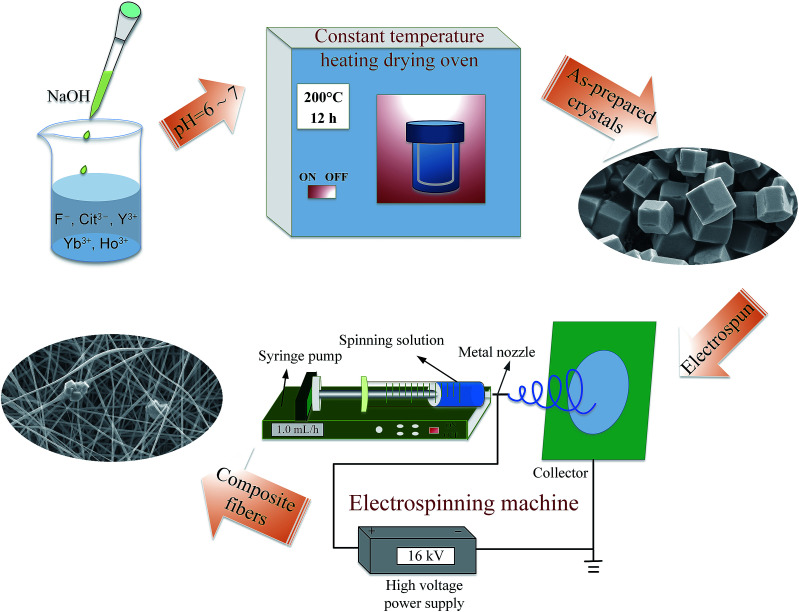
A diagram of NYF-HY/PAN composite fibers prepared *via* hydrothermal and electrospinning methods.

As-prepared NYF-HY MCs (0.1 g) were dispersed into the 11.5 g of *N*,*N*-dimethylformamide (DMF, AR), and then 1.0 g of polyacrylonitrile (PAN, *M*_w_ = 150 000) was slowly added into the solution with stirring for overnight to obtain a viscous spinning solution. The electrospinning instrument mainly includes a high voltage power supply, a collection device, a syringe pump and a syringe equipped with a metal nozzle, as shown in the electrospinning process illustrated in Fig. 1. In the electrospinning, the spinning sol was sprayed at a speed of 1.0 mL h^−1^ under a voltage of 16 kV. The distance between the tip of spinneret and the collector of aluminum foil was about 18 cm. A diagram of the process is shown in Fig. 1.

### 2.2 Characterization techniques

The crystal structure of the Yb^3+^/Ho^3+^ co-doped NaYF_4_ MCs was confirmed by X-ray diffraction (XRD) using a Shimadzu XRD-7000 diffractometer with Cu-Kα radiation operated at 40 kV and 30 mA. The morphology and the composition element analysis of the synthesized MCs and fibers were determined by using a Jeol JSM-7800F field-emission scanning electron microscope (SEM) and energy dispersive spectroscopy (EDS), respectively. The visible fluorescence spectra were captured on a Hitachi F-7000 fluorescence spectrophotometer using a 977 nm laser as the pumping source. When measuring the fluorescence, the excitation slit of the MCs was 1 nm, and the excitation slit of the composite fibers was 5 nm. The temperature-dependent UC luminescent spectra were measured with a Hitachi F-4600 fluorescence spectrofluorometer equipped with a 980 nm laser.

## 3. Results and discussion

### 3.1 Structural and morphological analysis

**Fig. 2:**
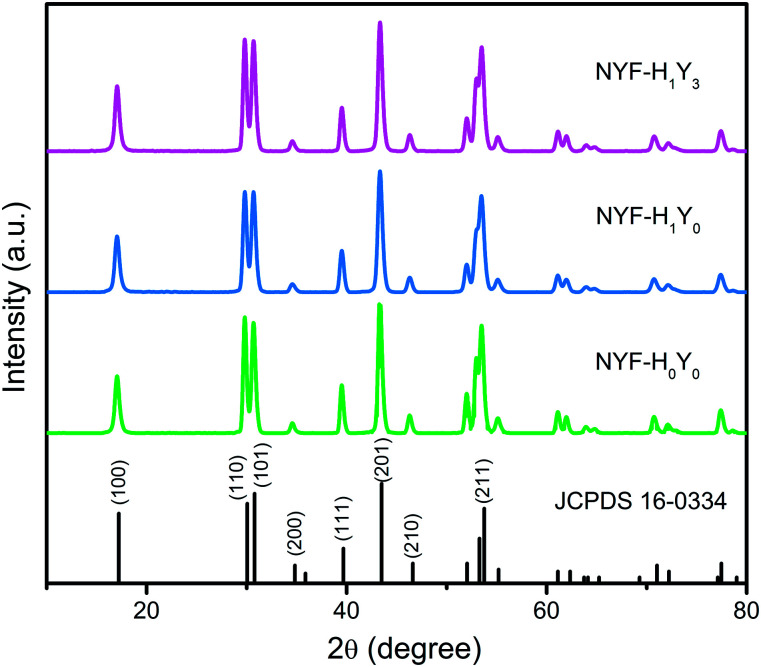
The XRD patterns of NYF-H_*m*_Y_*n*_ MCs with different Ho^3+^/Yb^3+^ doping concentrations.

**Fig. 3:**
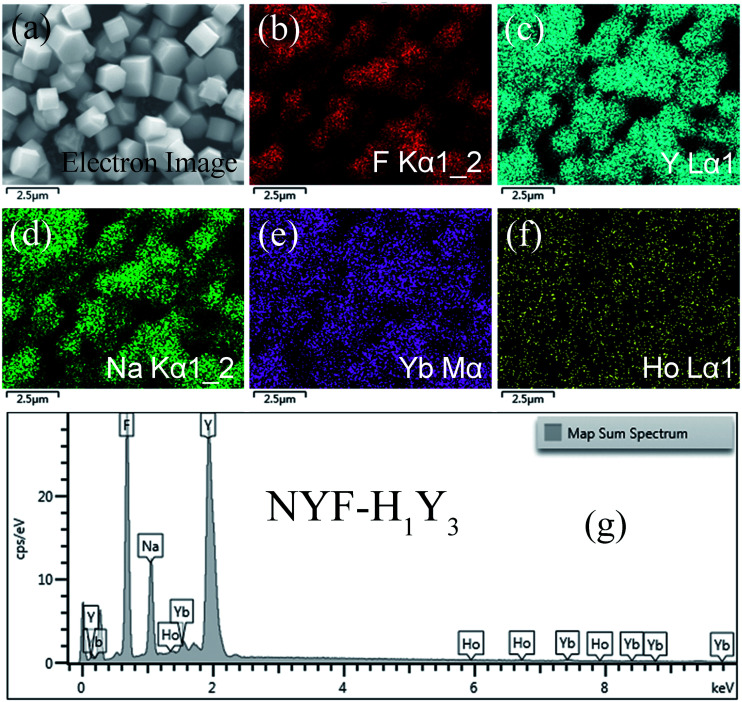
The (a–f) elemental mapping and (g) EDS spectrum of NYF-H_1_Y_3_ MCs.

The phase of the MCs was determined by XRD, as shown in Fig. 2. The data reported in JCPDS card no. 16–0334 (standard pattern of hexagonal NaYF_4_) and displayed in Fig. 2 (bottom part) was used to make a comparison with the XRD data of the prepared sample. It was seen that the diffraction peaks were sharp and clear indicating that the MCs were highly crystalline, and the diffraction peak of the MCs matched the NaYF_4_ standard card very well (space group *F*63*m*), demonstrating that the crystal structure and crystallinity after introducing Ho^3+^ and Yb^3+^/Ho^3+^ ions had not obviously changed. Fig. 3(a–f) show the elemental mapping spectra of the MCs, in which the elements of F, Ho, Na, Y, and Yb were clearly detectable and uniformly distributed. Furthermore, as shown in Fig. 3(g), in the representative EDS spectra of the corresponding region of Fig. 3(a), the F, Ho, Na, Y, and Yb peaks appeared. These results were consistent with the XRD results, indicating that Ho^3+^/Yb^3+^ co-doped NaYF_4_ MCs were successfully prepared by the hydrothermal method.

**Fig. 4:**
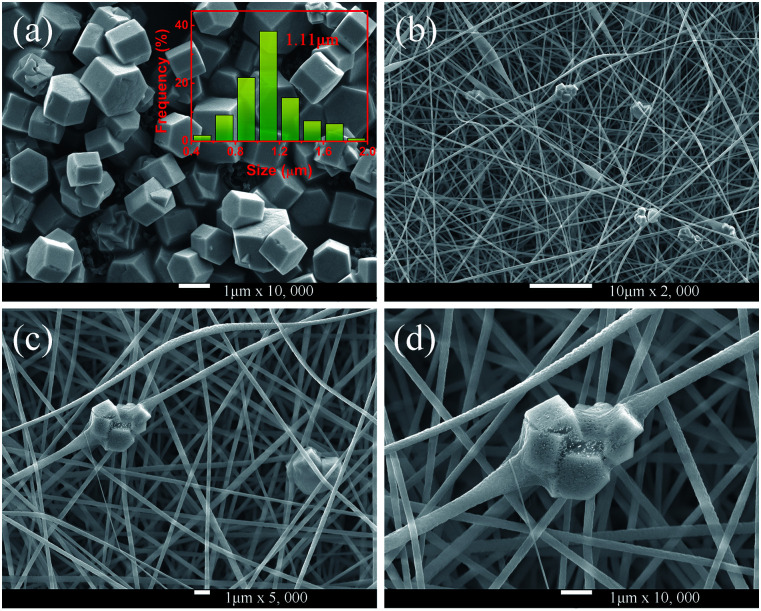
(a) SEM micrographs of NYF-H_1_Y_3_ MCs; the inset shows the corresponding size distribution. (b–d) SEM micrographs of NYF-H_1_Y_3_/PAN composite fibers at different magnifications.

As shown in Fig. 4, the morphologies of the MCs and nanofibers obtained were observed with SEM. Fig. 4(a) shows the morphological structure of the MCs as a hexagonal prism with a length and diameter of about 1 μm. Meanwhile, the size distributions of the MCs were counted, and the histogram of the particle size distribution is shown in the inset of Fig. 4(a). Moreover, a small number of crystals formed a mosaic structure, which might be because of the fact that twin crystals were connected to each other in order to reduce the surface energy in the process of crystal growth. Furthermore, Fig. 4(b–d) show the micrographs of the composite fibers at different magnifications, in which, fibers with a diameter of 300–500 nm and a smooth surface were arranged at random and overlapped with each other, in addition, several MCs were piled together to form a string of 3–4 μm beads and penetrated the fibers. The, crystals dispersed in the fibers had a larger surface area compared with the agglomerated MCs and the increased exposure area contributed to the full absorption of the infrared radiation.

**Fig. 5:**
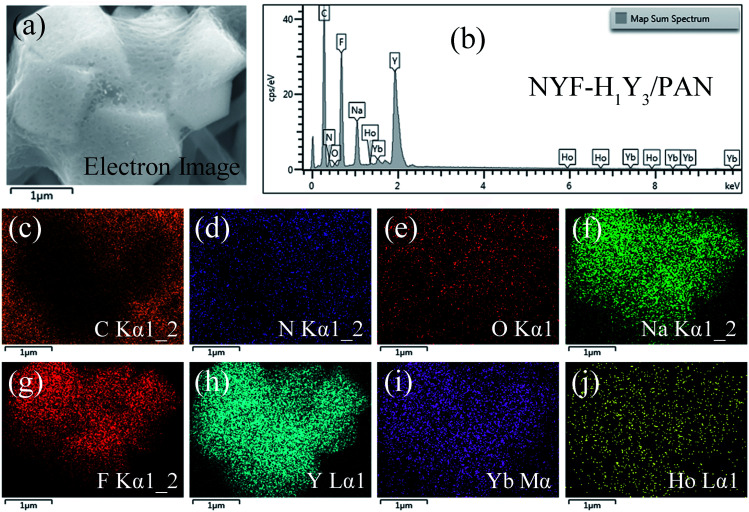
The (a, c–j) elemental mapping and (b) EDS spectrum of NYF-H_1_Y_3_/PAN fibers.

The composition element analysis of the NYF-H_1_Y_3_/PAN composite fibers was also carried out by EDS and the results are shown in Fig. 5. In the scanning area, a uniform distribution of the C, N and O elements derived from the polymer PAN and the solvent DMF can be found in the composite fibers. Furthermore, the distribution of F, Na, and Y elements was characterized by overall uniformity and local aggregation. The distribution of the Y element represented the location of Ho and Yb because of the integrity of the MC structure, therefore the distribution of Ho and Yb elements was in the same location as Y. The previous results were consistent with the results of the SEM analysis of the composite fibers, which proved that the hydrothermally synthesized MCs had been doped into the PAN fibers, and the composite fibers embedded with MCs had been prepared by electrospinning.

### 3.2 The UC luminescence properties of MCs and fibers

**Fig. 6:**
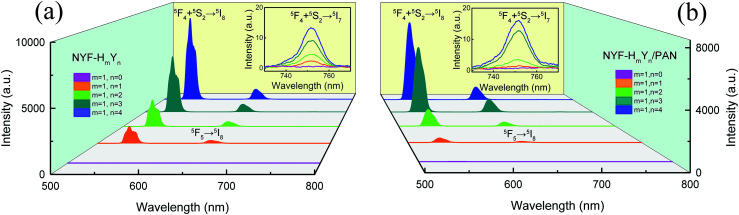
The UC spectra of (a) MCs and (b) composite fibers with different Yb^3+^/Ho^3+^ ion doping concentrations under 977 nm laser excitation. Insets: local magnification of the respective 752 nm emission peaks.

To investigate the effect of doping concentration on the UC emission fluorescence of the samples, the emission spectra of the MCs and composite fibers with different doping concentrations of Ho^3+^/Yb^3+^ were examined under the excitation of a 977 nm laser with 630 mW power, as shown in Fig. 6. The UC spectra of the MCs and composite fibers showed the same trends for the changes of the spectra. Under 977 nm excitation, a green emission band at 541 nm and two red emission bands at 646 and 752 nm were observed, which were attributed to (^5^F_4_, ^5^S_2_) → ^5^I_8_, ^5^F_5_ → ^5^I_8_, and (^5^F_4_, ^5^S_2_) → ^5^I_7_ transitions of Ho^3+^ ions, respectively. In addition, the co-doped concentration of Ho^3+^/Yb^3+^ ions has a great influence on the UC emission intensity, when the Yb^3+^ content is null (NYF–H_1_Y_0_ and NYF-H_1_Y_0_/PAN), and the photon energy of the 977 nm laser cannot effectively excite the Ho^3+^ ions, resulting in weak UC luminescence intensity. As the Yb^3+^ content increases, the intensity of each emission center increases significantly, and the bright UC luminescence can be observed, which demonstrated that there was an effective energy transfer between the Yb^3+^ and Ho^3+^ ions.

**Fig. 7:**
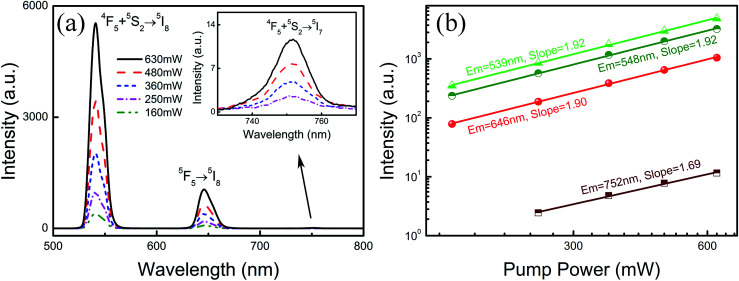
(a) The UC emission spectra of NYF-H_1_Y_3_/PAN composite fibers under 977 nm laser excitation at various powers. (b) The dependence of the UC emission intensities on excitation power in NYF-H_1_Y_3_/PAN composite fibers.

In order to investigate the frequency conversion emission of NYF-H_1_Y_3_/PAN flexible fibers, the dependence of the UC spectra on the pump power was determined, and the results are shown in Fig. 7(a), which demonstrated that the green and red UC emissions of Ho^3+^ could be effectively excited by 977 nm lasers. To further explain the UC multi-photon excitation mechanism of the composite fibers, the excitation power dependence of the green and red UC emission fluorescence is shown in Fig. 7(b). It is well known that UC emission intensity (*I*) relies on the pump power (*P*), which follows *I* ∝ *P*^a^, where *a* is the number of pump photons participating in the UC process.^52–54^ Using linear fitting, the *a* values of the emission peaks at 539, 548, 646 and 752 nm were 1.92, 1.92, 1.90 and 1.69, respectively, which indicated their participation in the two-photon process.

**Fig. 8:**
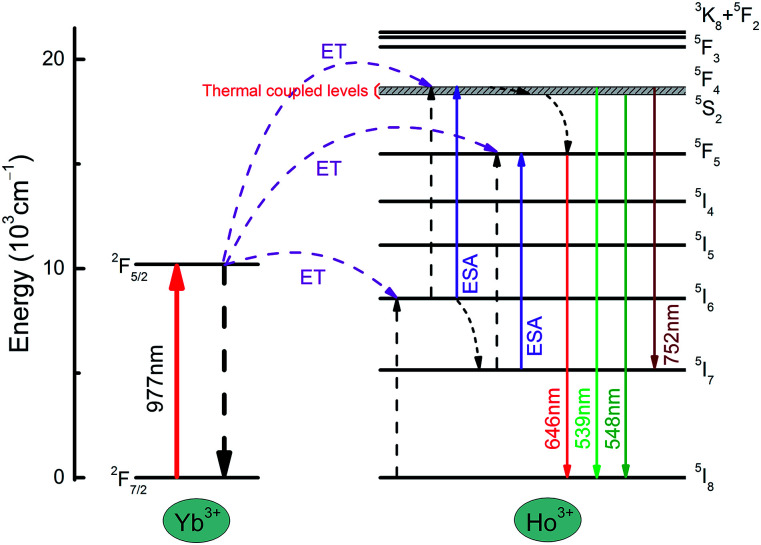
A schematic energy level diagram of the luminescence mechanism of NYF-HY/PAN fibers under laser excitation at 977 nm.

The energy transfer mechanism of the NYF-HY/PAN composite fibers is shown in Fig. 8. First and foremost, under the excitation of 977 nm, the Ho^3+^ ions in the ground state ^5^I_8_ level are used to fill the excited state ^5^I_6_ level by energy transfer (ET) from the adjacent Yb^3+^ ions in excited state ^2^F_5/2_ energy level. Some Ho^3+^ ions at the ^5^I_6_ level relaxed to the excited state ^5^I_7_ by non-radiative relaxation processes (NRP). Subsequently, the excited state of Ho^3+^ further absorbed energy to the ^5^F_4_ and ^5^F_5_ levels by excited state absorption (ESA) or ET processes, and then returned to the ground state to form 539 and 646 nm emission, respectively. Meanwhile, a part of Ho^3+^ at the ^5^F_4_ level by NRP relaxed to ^5^S_2_ and ^5^F_5_, and then transited to the ground state, producing emission bands of 548 and 646 nm. Therefore, there were two transition pathways for the red emission at 646 nm. In addition, the transition from the ^5^F_4_/^5^S_2_ energy level to the ^5^I_7_ energy level produced an emission band of 752 nm. Hence, the emission bands of the composite fibers are both two-photon processes, which was consistent with the dependence of the UC luminescence spectrum on pump power.

### 3.3 Optical thermosensitive properties of MCs and fibers

**Fig. 9:**
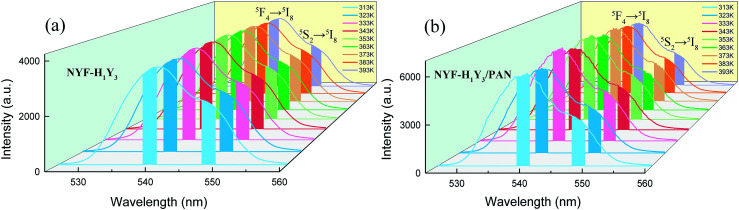
The UC spectra of (a) NYF-H_1_Y_3_ MCs and (b) NYF-H_1_Y_3_/PAN fibers at 980 nm excitation with 400 mW power from 313 to 393 K.

The green UC spectra of NYF-H_1_Y_3_ MCs and NYF-H_1_Y_3_/PAN composite fibers were excited by a 980 nm laser with a power of 400 mW at different temperatures, as shown in Fig. 9. With the increase of temperature, whether it is MCs or fibers, the peak positions of the green UC emission at 539 and 548 nm had not changed, but their UC intensities and FIR value had changed. The thermally coupled ^5^F_4_ and ^5^S_2_ energy levels of Ho^3+^ were near to each other, and their overall distribution agrees with the Boltzmann distribution law.^55^ The FIR of the green UC emission at 539 and 548 nm can be expressed as:^56,57^1
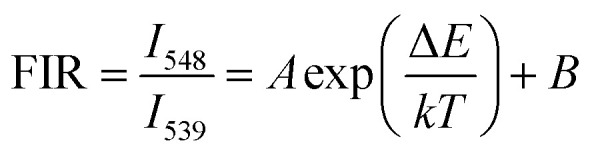
where *I*_539_ and *I*_548_ are the integrated intensities of the ^5^F_4_ → ^5^I_8_ and ^5^S_2_ → ^5^I_8_ transitions, respectively. *A* and *B* are the fitting coefficients, Δ*E* is the energy gap separating the two excited states, *k* (0.695 cm^−1^ K^−1^) is the Boltzmann constant, and *T* is the absolute temperature. Clearly, the value of the FIR is only related to the variable *T*, which can accurately reflect the temperature change of the system.

**Fig. 10:**
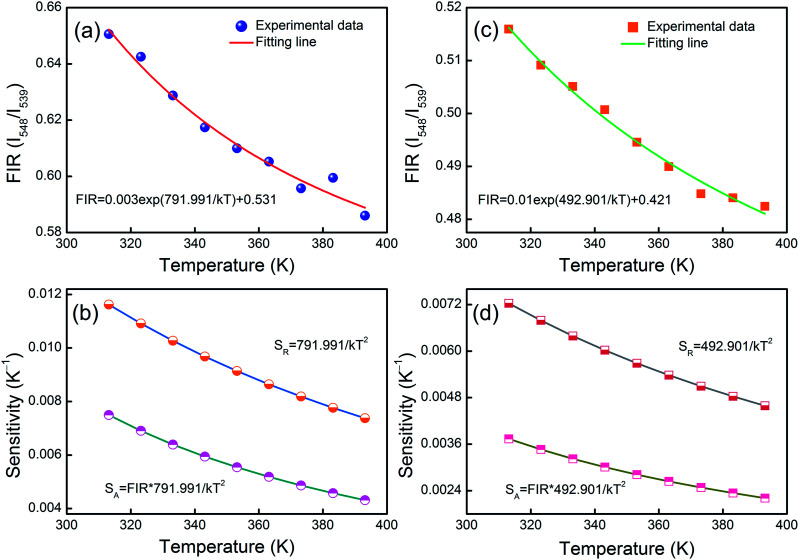
(a) The relationship between *I*_548_/*I*_539_ and temperature and (b) plots of the sensitivity of (*I*_548_/*I*_539_) from the NYF-H_1_Y_3_ MCs. (c) The relationship between *I*_548_/*I*_539_ and temperature and (d) plots of the sensitivity of (*I*_548_/*I*_539_) from the NYF-H_1_Y_3_/PAN composite fibers.

Fitting the experimental data with eqn (1), *A*_1_ = 0.003, *B*_1_ = 0.531, Δ*E*_1_ = 791.991 for the MCs,*A*_2_ = 0.010, *B*_2_ = 0.421 and Δ*E*_2_ = 492.901 for the composite fibers, were obtained. The FIR of the green UC emission at 539 and 548 nm of the MCs and composite fibers *versus* temperature in the range of 313–393 K are shown in Fig. 10(a) and (c). Additionally, the absolute sensitivity *S*_A_ and relative sensitivity *S*_R_ are important evaluation criteria for optical thermometry applications, which can be calculated as:2
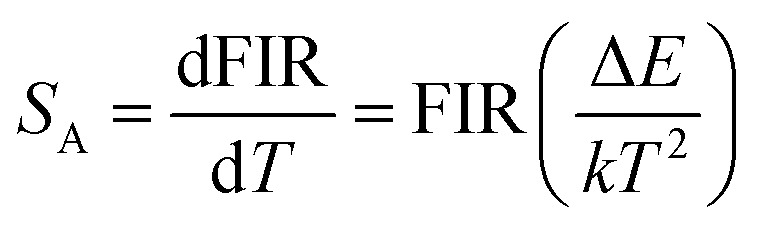
3
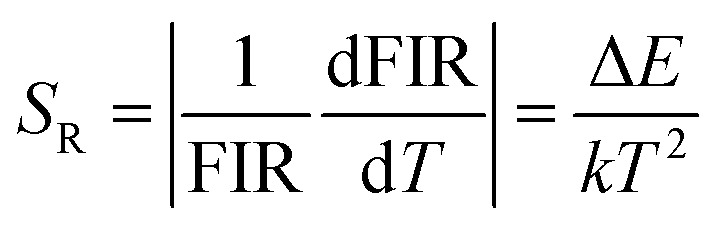


The sensitivity curves of the MCs and composite fibers are shown in Fig. 10(b) and (d), respectively. It can be clearly seen that the sensitivity curves of the MCs and composite fibers have the same trend, that is, the *S*_A_ and *S*_R_ of the MCs and composite fibers decreases with the increase of temperature in the temperature range of 313–393 K. At 313 K, the *S*_A_ values of the MCs and composite fibers reached 0.0075 K^−1^ and 0.00373 K^−1^, respectively, and correspondingly, the *S*_R_ values reached 1.162% K^−1^ and 0.723% K^−1^. For comparison, the maximum sensitivity *S*_A_ and *S*_R_ of some temperature sensor materials based on doped Ho^3+^ at different temperature ranges (Δ*T*) and diverse excitation wavelengths (*λ*_ex_) are listed in Table 1, and the sensitivity error (Δ*S*) of the composite fibers is also shown in the table. Compared with other Ho^3+^ doping temperature sensing materials, the Ho^3+^/Yb^3+^ co-doped materials had an intense UC fluorescence emission and great temperature sensing performance under the 980 nm excitation, which was due to the fact that the energy levels of Yb^3+^ and Ho^3+^ matched better to facilitate the energy transfer. In this work, NYF-H_1_Y_3_/PAN composite fibers had high sensitivity at low temperatures, and more importantly, its external shape and color were almost unchanged at the measurement temperature, which indicated that flexible composite fibers had potential prospects for applications in biological temperature measurement.

**Table 1:** Maximum sensitivity *S*_A_ and *S*_R_ values for temperature sensor materials in different temperature ranges

Research object	Transitions	*λ*_ex_	Δ*T* (K)	*S*_A_ (K^−1^)	Δ*S* (10^−3^ K^−1^)	*S*_R_ (% K^−1^)	Reference
Y_2_Ti_2_O_7_:Yb, Ho	^5^F_4_/^5^S_2_, ^5^F_5_ → ^5^I_8_	980 nm	423–693	0.0018	—	1328.17/*T*^2^	58
LaNbO_4_:Nd, Yb, Ho	^5^F_4_/^5^S_2_, ^5^F_5_ → ^5^I_8_	808 nm	303–693	0.00204	—	—	26
Y_2_O_3_:Zn, Yb, Ho	^5^F_3_, ^3^K_8_ → ^5^I_8_	980 nm	299–673	0.00302	—	1067.76/*T*^2^	59
In–Zn–Sr–Ba:Ho	^5^F_4_/^5^S_2_ → ^5^I_8_, ^5^I_7_	473 nm	20–300	0.0036	—	—	60
NaYF_4_:Yb, Ho/PAN	^5^F_4_, ^5^S_2_ → ^5^I_8_	980 nm	313–393	0.00373	0.0123	0.723	This work
Y_2_O_3_:Ge, Yb, Ho	^5^F_4_/^5^S_2_, ^5^F_5_ → ^5^I_8_	976 nm	300–400	0.0052	—	0.62	8
ZnWO_4_:Yb, Ho	^5^F_4_/^5^S_2_ → ^5^I_8_, ^5^I_7_	980 nm	83–503	0.0064	—	—	10
NaYF_4_:Nd, Ho	^5^F_4_/^5^S_2_, ^5^F_5_ → ^5^I_8_	808 nm	308–473	—	—	0.90	42

## 4. Conclusions

In summary, NYF-HY MCs and NYF-HY/PAN composite fibers were prepared *via* hydrothermal synthesis and electrospinning, respectively. It is confirmed that the hexagonal structure and thermally reactive functionality of the embedded crystals in the fibers are preserved. The green fluorescence emission from thermal correlation levels ^5^F_4_ and ^5^S_2_ of Ho^3+^ was verified, and the fluctuation of the green emission intensity ratio was studied as a function of temperature in the range of 313–393 K. Moreover, the thermosensitive sensing behavior of NYF-HY/PAN flexible fibers is investigated *via* the FIR technique, and the maximum absolute and relative sensitivity values for the present materials are 0.00373 K^−1^ at 313 K and 0.723% K^−1^ at 313 K, respectively. The NYF-HY/PAN composite fibers with the excellent properties of flexibility, sensitivity, and stability can be adopted as sensing materials for biological temperature measurements.

## Author contributions

Yan Zhang carried out the experiments, fabricated the temperature sensing materials, and wrote the manuscript. Zelin Gao and Yue Li helped to rewrite the manuscript during the revision process. Hai Lin and Edwin Yue Bun Pun provided guidance and supervision. All the authors have read and approved the final manuscript.

## Conflicts of interest

There are no conflicts to declare.

## Supplementary Material
